# How to Control Subannular Hemorrhage during Aortic Root Enlarging Procedures

**DOI:** 10.1055/s-0039-1683387

**Published:** 2019-04-01

**Authors:** Abdallah K. Alameddine, Brian Binnall, Frederick T. Conlin, Khaled O. Alameddine

**Affiliations:** 1 Division of Cardiac Surgery, Baystate Medical Center, Springfield, Massachusetts; 2 Division of Anesthesiology, Baystate Medical Center, Springfield, Massachusetts; 3 Department of Internal Medicine, Swedish Covenant Hospital, Chicago, Illinois

**Keywords:** aortic stenosis, small aortic root, destroyed aortic annulus, subannular patch bleeding, aortoventricular rupture

## Abstract

We describe an effective suture technique to control the persistent subannular bleeding at the aortoventricular curtain in four patients with aortic stenosis and small annulus who underwent aortic root enlargement and patch reconstruction. This technique approximates the left atrial roof to the aortic root without the need for re-replacement of the aortic prosthesis or revision of the patch. Reintervention for aortic root, valve, or the residual aorta was not required.

## Introduction

Ongoing subannular bleeding (SAB) following aortic root enlargement can be life threatening and justifies an aggressive surgical posture. In the case of aortic root enlarging procedures with a patch repair (Nicks or Manougian incisions in patients with a small aortic annulus), 1 2 intraoperative bleeding seeping through the anchoring lower end of the patch repair is most likely to occur following extensive annular debridement, excessive suture tension for tissue approximation (resulting in disruption of friable tissue), inaccessible exposure due to the small aortic root, or implantation of an oversized prosthesis. The bleeding site at the aortoventricular curtain cannot be visualized due to the narrow anatomic space as it is subannular under the prosthetic valve sewing ring (whether it is mechanical or bioprosthetic) 3 and risks becoming life threatening.

Various approaches to hemorrhage control at this site have been attempted. One traditional option entails redoing the entire procedure and replacing the old prosthesis and the patch, which can significantly prolong ischemic and bypass time. The other option, as we previously reported, is a “U” stitch repair to control bleeding from a damaged intertrigonal area (aortic–mitral curtain) during tissue aortic valve replacement (AVR) by reinforcing the subannular tissue from within the aorta without removing the aortic bioprosthesis. 3 However, this technique cannot be used when dealing with a mechanical aortic prosthesis.

In the current approach, the left atrial dome tissue is approximated to the neighboring greater curvature of the aorta with the goal being complete obliteration of the subannular aortoventricular bleeding site. This method has been used successfully in a small clinical series of four patients without the need for removal of the old prosthesis or revision of the patch.

## Patients and Methods

### Patient Profiles and Clinical Experience

Between December 2014 and April 2016, four patients underwent AVR with patch enlargement procedures for small aortic root complicated by intraoperative SAB and were retrospectively reviewed. Waiver for individual consent was granted by our Institutional Review Board. The demographic and clinical patient characteristics as well as outcomes data are reported in Table 1 . The ages of the patients were between 59 and 78 years. Before the operation, all patients were investigated by echocardiography and cardiac catheterization. Postoperatively, patients were evaluated by clinical examination and echocardiography.

**Table 1:** Demographic and clinical patient characteristics and outcomes data

Variables	Case 1	Case 2	Case 3	Case 4
Age	78	77	59	77
Sex	F	M	F	M
Primary diagnosis	Aortic stenosis	Aortic and mitral stenosis	MSSA infective endocarditis with root abscess	Aortic stenosis 5.5 cm Ascending aortic aneurysm
Operation performed	AVR (#21 St. Jude Trifecta) Aortic root enlargement with Hemashield patch	Redo AVR (#19 St. Jude Regent) MVR (#19 Magna Ease) and enlargement with Hemashield patch	Redo AVR (#19 St Jude Regent), bovine patch reconstruction, LVOT with aortic annular enlargement	AVR (#29 Model 2700), Ascending aortic replacement (#30 Hemashield tube graft and patch) Left atrial Cryo-Maze, left atrial appendage ligation
24-hour chest tube output	360 mL	430 mL	75 mL	320 mL
Re-exploration for bleeding	No	No	No	No
Outcome	Alive and well after 14 months follow-up	Alive with liver cirrhosis after 28 months follow-up	Died POD #23, MSOF secondary to right heart failure.	Alive with metastatic pancreatic cancer after 10 months follow-up
Postop aortic gradient (mm Hg)	15	20	12	6
Cross clamp time/CPB time (min)	107/149	341/424	203/226	190/200

Abbreviations: AVR, aortic valve replacement; CPB, cardiopulmonary bypass; LVOT, left ventricular outflow tract; MSOF, multiple system organ failure; MSSA, methicillin-sensitive Staphylococcus aureus; POD, postoperative day.

### Surgical Technique

This technique might be applicable in all types of SAB including modified Bentall procedures. In AVR, a median sternotomy and cardiopulmonary bypass (CPB) are used in a standard fashion. Usually the bleeding is noticed immediately when coming off CPB originating from below the valve. The following repair technique is used preferably with reinstating CPB to improve visualization and to prevent further injury to the aorta or left atrial dome.

**Fig. 1:**
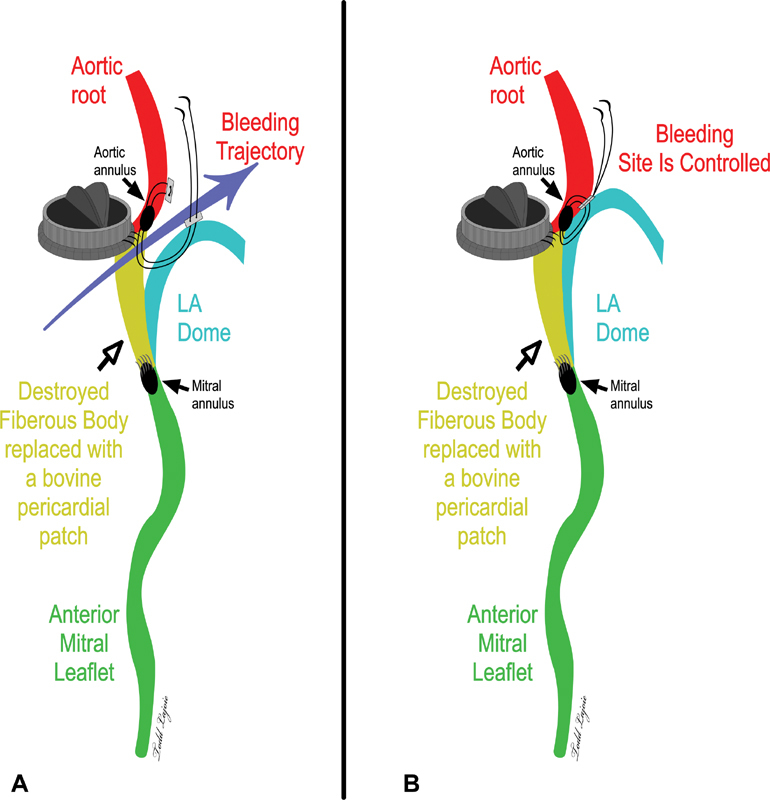
Illustration of the surgical repair: ( **A** ) before: the bleeding in such a situation happens at the *lowermost point* of the patch and ( **B** ) after placement of the stitch (es) *above* the bleeding site. LA, left atrium.

Full-thickness bites are made through the left atrial dome myocardial tissue and are passed through to the neighboring aortic root tissue, closing the gap by using pledgeted double-armed renal artery bypass (RB) 1 or small half circle (SH) 4–0 polypropylene sutures. It is also possible to use 2-0 Ethibond (Ethicon, Sommerville, NJ) polyester sutures. The bites on the aortic wall should be periadventitial (partial thickness) to reduce the theoretical risk of transmural aortic injury with subsequent pseudoaneurysm formation. The other arm of the suture is brought back through the dome and tied to the first suture arm. Teflon felt pads are used for reinforcement of the suture line and to avoid cutting through and disrupting this thinned area. Knot tying is done snug, just enough to exclude the space by attaching (imbricating) the left atrial dome tissue superiorly against the exposed outer aortic wall caudally. Further sutures may be required by carrying the suture line along the left side of the aorta and going over the upper adventitial edge of the right pulmonary artery and sealing off the right entrance of the transverse sinus; any potential exit for the bleeding is thus sealed. The additional sutures are spaced 2 mm apart using the prior meticulously placed suture to retract, as one goes around the suture line closure from right to left. The final result is illustrated in the Fig. 1 , where the bleeding trajectory—originating from the lower end of the patch—is sealed off superiorly. During placement of the sutures, CPB flow is decreased but keeping up with the left ventricular vent to avoid sucking air from the suture needle holes in the left atrial dome.

## Results

Mean age of patients was 68 years and two were female. A modified Manouguian technique was performed without entering into the left atrium. A limited incision over the anterior mitral leaflet was done for a distance < 4 mm, and the enlargement was achieved by extending the aortotomy posteriorly into the fibrous tissue between the noncoronary cusp and the left coronary cusp and onto the subaortic curtain. One patient had the aortic valve replaced with a mechanical prosthesis and three with a bioprosthetic valve. The choice of patch was the surgeon's preference. Concomitant procedures included mitral valve replacement in one patient, and ascending aortic aneurysm repair and a CryoMaze procedure in another. Redo AVR was performed in two patients, in one of whom Staphylococcus aureus endocarditis was present. Two patients received a mechanical prosthesis (St. Jude Medical, St. Paul, MN) and two patients received bioprosthesis (Edwards Lifesciences, Irvine, CA). There was one hospital death from multisystem organ failure; the remaining three patients were well at last follow-up visit, and there has been no late death. All of the patients underwent transthoracic echocardiography with a mean postoperative interval of 16 months, and none required reintervention for aortic root, the valve, or for the residual aorta.

## Discussion

There is naturally a weakened and thinned out area where the mitral valve and the subaortic curtain meet between the fibrous trigones that separate it from the outside. 4 Intraoperative SAB from disruption of this friable area, following AVR with patch enlargement of the root, is a major and severe complication with an unknown incidence.

In a retrospective review of 2,366 cases of AVR performed at the Mayo Clinic over a 9-year period, 10.5% of all AVRs required a posterior aortic root enlargement. In the root enlargement group, a 4.8% rate of reoperations for bleeding occurred (unspecified bleeding site), and the reported raw operative mortality was 5.6%. 5

### Prevention of Bleeding

Our recommendation is not to disrupt the thin and delicate layer of loose tissue veil that attaches the left atrial dome to the aortic root during the dissection in preparation for the enlargement of the aortic root. This step of the operation should be done meticulously using a Kittner dissector or the tips of scissors to free just enough of the aortic root from its surrounding tissues staying on the left atrial wall. The extra time spent on this step of the operation will pay great dividends by avoiding pseudoaneurysm formation in the aortic root, as well as bleeding into and separation of the left atrial wall. 6 7

We reserve the current approach for cases in which the tear or dehiscence is localized or confined to a single area. It can also be used as a prophylaxis before the aortic cross-clamp is released if the repair was thought to be untenable. The main advantages are retaining the prosthesis in place and avoiding longer ischemic and bypass time. If bleeding recurs after the stitch is placed, then that situation is addressed by redoing the entire procedure.

In conclusion, the current extra-aortic stitch is the suggested management option in selected individual patients with unexpected SAB when reclamping the aorta is undesirable. This choice is a potentially very useful technique, and it offers a less invasive and more facile technique given the alternative choice of redoing the entire valve replacement procedure with direct access via an intra-aortic approach and recross-clamping the aorta. Further studies with a larger numbers of patients and longer follow-up are required to validate this approach.
